# Long-term nutritional management of an obese German Spitz with paroxysmal dyskinesia, calcium oxalate urolithiasis, and suspected pancreatitis—A case report

**DOI:** 10.3389/fvets.2023.1054251

**Published:** 2023-03-14

**Authors:** Camila Baptista da Silva, Michelle Hermans, Norberto Ruiz-Suárez, Fien Verdoodt, Sofie Fatima Mareyam Bhatti, Myriam Hesta

**Affiliations:** ^1^Department of Morphology, Imaging, Orthopaedics, Rehabilitation and Nutrition, Ghent University, Merelbeke, Belgium; ^2^Department of Small Animal, Faculty of Veterinary Medicine, Ghent University, Merelbeke, Belgium

**Keywords:** dog, nutrition, neurological signs, gastrointestinal signs, bladder stones

## Abstract

**Background:**

To our knowledge, this is the first description of long-term nutritional management in a dog with paroxysmal dyskinesia.

**Case summary:**

An obese 9-year-old, male entire, German Spitz was presented for dietary management after being diagnosed with calcium oxalate urolithiasis and suspected pancreatitis. Since he was seven years old, the dog has had a history of neurological signs, which were thought to be epileptic seizures. He was treated with phenobarbital and potassium bromide and was clinically controlled. For his nutritional advice, aiming to minimize one of the most important risk factors for the diseases, a weight loss program was started and successfully executed. However, 10 months later, the dog restarted presenting neurological episodes at a high frequency (3x/week). Based on videos and the characteristics of the neurological signs, the dog was diagnosed with paroxysmal dyskinesia. To investigate the role of gluten intake on this patient's neurological signs, a dietary trial with a commercial hypoallergenic diet (gluten-free; hydrolyzed protein) was followed. During the 3 months of the dietary trial, four neurologic episodes related to food indiscretion occurred. Upon the decrease in neurological episodes, the anti-seizure drugs were slowly discontinued. During this period, the dog presented only two neurologic episodes that were related to the days that the anti-seizure drugs were decreased. For 4 months the dog remained episode-free. However, a change in the dog's diet to another gluten-free diet (higher fat) led the dog to vomit and experience another neurologic episode. Once the dog was back to the previous gluten-free diet, it clinically improved, and no other clinical signs were reported by the client during the next 5 months.

**Conclusion:**

Although a relationship between gluten and paroxysmal dyskinesia cannot be confirmed, the dog's improvement after the nutritional management and the removal of the anti-seizure therapy is supportive of dietary association.

## 1. Introduction

Canine paroxysmal dyskinesia (PD) is a movement disorder characterized by recurrent episodes of abnormal and involuntary self-limiting movements (1). Although it is not a life-threatening disease, the neurologic episodes can last minutes or hours (1) and may be distressing for the owners.

Over the last few years, researchers have been studying possible dietary causes of PD (2–6). Some case reports have shown clinical improvement in Border-Terriers after being fed gluten-free diets (4, 6). However, long-term nutrition follow-up has never been reported. The focus of the presented case is the nutritional management of a patient with multiple diseases: PD, obesity, calcium oxalate (CaOx) urolithiasis, and possible pancreatitis.

## 2. Case description

**Figure 1:**
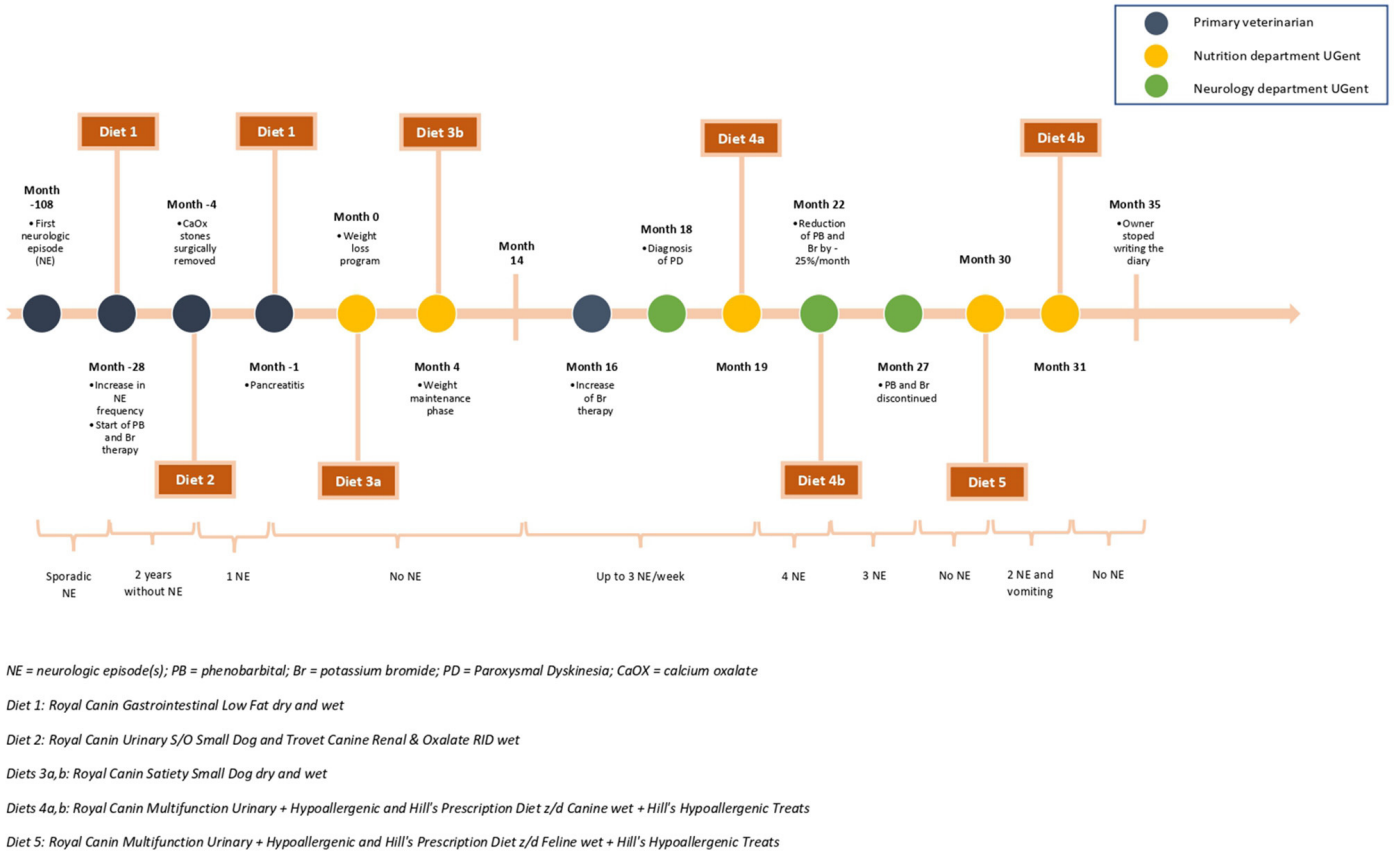
Timeline showcasing most important events, neurological episodes (NE), anti-seizure drugs and dietary changes.

A 9-year-old, male entire, German Spitz was first presented to its regular veterinary surgeon with lower urinary tract symptoms (Month -4). Bladder stones were detected and surgically removed. The stones were sent to an external laboratory and were diagnosed as CaOx (10% CaOx monohydrate and 90% CaOx dihydrate). The dog's medical history also indicated occasional neurological episodes since it was 3 months old (Month -108), which were thought to be epileptic seizures. By the age of seven years (Month -28), the frequency of the neurological episodes increased but was controlled with phenobarbital (7 mg/kg ideal body weight [iBW] BID) and potassium bromide (23 mg/kg iBW BID) as anti-seizure drugs (ASD). The dog was episode-free for 2 years (Figure 1). Due to the use of the medication and its increased-related risk of pancreatitis (7–9), commercial wet and dry low-fat diets (diet 1; estimated total daily fat intake: 2.4 g/kg^0.75^) were given until CaOx stones were diagnosed. The dog was also occasionally fed rice and yogurt as a treat. After removal of the CaOx stones, the dog was put on dry and wet diets designed to prevent uroliths (diet 2; estimated daily fat intake: 5.8 g/kg^0.75^) (Month -4).

Three months later (Month -1), the dog was hospitalized due to abdominal pain, and gastrointestinal and neurologic complaints. The owner reported that since diet 2, the dog had low fecal consistency and darker feces color. Blood work revealed mild anemia, high serum alkaline phosphatase (406 U/L; reference range 23–212 U/L), serum amylase (>2,500 U/L; reference range 500–1,500 U/L), and serum lipase (>6,000 U/L; reference range 200–1,800 U/L) values. As pancreatitis was suspected, low-fat dry and wet diets were resumed (diet 1). The dog was then referred to the Faculty of Veterinary Medicine of Ghent University for its nutritional management (Month 0).

On initial physical examination, the dog was weighing 4.4 kg and had a body condition score (BCS) of 8/9 with an estimated iBW of 3.4 kg. Cranial abdominal pain and mild periodontitis were noticed. The dog was fed a mixture of 100 grams of wet food per day and *ad libitum* dry food. The wet food was given as a meal and also to facilitate the dog's medication intake.

Considering the dog's physical examination, clinical and feeding (Table 1) history, risk factors (10, 11) (Table 2), and key nutrients (10) (Table 3) for each disease (obesity, chronic pancreatitis, and CaOx), a weight loss regimen was advised. The selection of the diet aimed to prevent CaOx by feeding a low relative supersaturation (RSS) diet, prevent pancreatitis by reducing the fat intake, and safely reduce the dog's body weight (BW). The prescribed weight loss diet (diet 3a; 2.4 g fat/kg^0.75^) was given at 70kcalxkg iBW^0.75^ and consisted of a mixture of commercial dry and wet food and, upon the owner's request, two options of vegetables (zucchini or cucumber) (5% energy intake) were advised to be fed in a food dispenser toy. Four months later (Month 4), the dog weighed 3.5 kg and had a BCS of 5/9 and the mean weight loss rate was determined at 1.72% of BW per week. To keep the dog at a stable weight, the energy intake was then slowly increased to up to 15% (diet 3b; 2.8 g fat/kg^0.75^), and the dog maintained its weight without presenting gastrointestinal, neurological, or low urinary tract symptoms hereafter.

**Table 1:** Comparison between key nutrients (expressed in g/kg^0.75^) and ingredients of the patient's diets throughout its life.

	**Nutrient levels (adult dogs)**			**Advised diets by primary veterinarian**		**Advised diets by the nutritional service from the University of Ghent**				
	**Minimum by NRC**	**Recommended by NRC**	**Recommended by FEDIAF**	**Diet 1^a^**	**Diet 2^b^**	**Diet 3a^c^**	**Diet 3b^c^**	**Diet 4a^d^**	**Diet 4b^d^**	**Diet 5^e^**
kcal ME/kg^0.75^			80	120^*^	120^*^	71	81	81	78	78
kJ ME/kg^0.75^			335	500^*^	500^*^	296	341	341	326	325
**Nutrients (g/kg** ^0.75)^										
Protein	2.62	3.28	4.95	8.34	6.38	8.87	10.07	4.41	4.20	5.11
Fat		1.80	1.51	2.36	5.79	2.41	2.79	3.28	3.13	3.51
Calcium	0.06	0.13	0.14	0.36	0.21	0.21	0.24	0.13	0.13	0.13
Phosphorous		0.10	0.11	0.25	0.18	0.16	0.19	0.11	0.11	0.12
Sodium	0.01	0.03	0.03	0.13	0.27	0.08	0.10	0.23	0.19	0.24
Magnesium	0.01	0.02	0.02	35.53	13.50	0.19	0.22	10.71	0.11	0.10
**Other**										
Gluten-containing ingredient				Wheat and grains	Wheat	Wheat and grains		No		No
Urinary claim				-	Low RSS and pH-increasing effect disclaimer	Low RSS		Low RSS		Low RSS
Hypoallergenic with hydrolyzed protein				No	No	No		Yes		Yes

Due to different energy intakes over time, the evaluation of the diets was made in g/kg^0.75^.

^*^Estimated energy intake based on FEDIAF, 2021 for > 7-year-old adult dog.

^a^Royal Canin Gastrointestinal Low Fat dry and wet.

^b^Royal Canin Urinary S/O Small Dog and Trovet Canine Renal & Oxalate RID wet.

^c^Royal Canin Satiety Small Dog dry and wet.

^d^Royal Canin Multifunction Urinary + Hypoallergenic and Hill's Prescription Diet z/d Canine wet + Hill's Hypoallergenic Treats.

^e^Royal Canin Multifunction Urinary + Hypoallergenic and Hill's Prescription Diet z/d Feline wet + Hill's Hypoallergenic Treats.

**Table 2:** List of risk factors for each associated disease that were taken into account for this patient's nutritional recommendation.

	**Obesity**	**Calcium oxalate stones**	**Chronic pancreatitis**	**Paroxysmal dyskinesia**
Drug	Treatment with phenobarbital		Treatment with phenobarbital	Treatment with phenobarbital
Medical conditions		Obesity	Obesity	
		Chronic pancreatitis		
Animal odds		Gender: male	Male	
		Breed predisposition (4%)		
		Age: older (8.5 average)	Age: older	The onset of symptoms at an early age (< 1 year)
Dietary factors	High fat	Homecooked diet	High fat	Possible correlation to gluten-containing diets
	High energy dense diets	High protein	Low protein	
	Free-choice feeding	High sodium, vitamin D, and magnesium: hypercalciuria		
	Treats	High vitamin C: Hyperoxaluria		
		Deficiency of pyridoxine: Hyperoxaluria		
		Low magnesium, phosphorous, and calcium		

Hand et al. (10).

Lowrie and Garosi (11).

**Table 3:** Key nutrients and their aimed values for the associated diseases.

**Key nutrients**	**Obesity**	**Calcium oxalate stones**	**Chronic pancreatitis**	**Paroxysmal dyskinesia**
Energy density	< 3.4 kcal/g or 14.2 kJ/g	-	-	-
Water	Increased	High: to decrease urine supersaturation	High: due to dehydration	-
Protein	High: >25% DM	Avoid excess: 10–18% DM	Moderate to low: 15–30% DM	Hydrolyzed; gluten-free
Fat	Low: < 9–14% DM	-	Low: < 10–15% DM	
Fiber	High: 12–25% DM	High: to decrease intestinal absorption and urinary excretion of Ca	-	-
Calcium (Ca)	-	Avoid excess: 0.4–0.7% DM	-	-
Phosphorous (P)	0.4–08% DM	Avoid deficiency: 0.3–0.6% DM	-	-
Ca:P		Ratio: 1.1:1–2:1	-	-
Sodium	0.2–04% DM	Moderate restriction: < 0.3% DM	-	-
Magnesium		Moderate: < 0.04–0.15% DM	-	-
Vitamin C	> 100 mg/kg	Avoid supplements and food containing vitamin C	-	-
L-carnitine	>300 ppm	-	-	-
Oxalate	-	Avoid high oxalic acid sources	-	-

%DM, percentage of dry matter.

Hand et al. (10).

Ten months later (Month 14), the neurological signs increased to three episodes per week and were unresponsive to a higher dose of potassium bromide (23 mg/kg iBW in the morning and 46 mg/kg iBW in the evening), the dog was then referred to the neurology service of the same Faculty (Month 18). The episodes were mainly occurring during rest and lasted for approximately one to 3 min. During the episode, the dog was conscious and able to respond to its owner calling its name, but the episodes could not be stopped by distraction. Videos of the episodes (Supplementary material) showed dystonia-like movements (stiffness and cramping of ≥1 muscle group) mainly of the limbs and neck. No autonomic signs were reported. After an episode, the dog was immediately normal again. The first episode was often followed by a second one within minutes. Clinical and neurological examination revealed no abnormalities and recent blood tests did not show metabolic causes for the increased number of episodes. In addition, serum concentrations of ASDs were constantly monitored over the years and were within therapeutic levels. Based on the videos of the neurological episodes, PD was suspected. Brain MRI was not performed as the dog was normal in-between episodes for ≥6 years, suggesting no structural brain disease. Aiming to investigate the relationship between diet and neurological episodes, an elimination diet trial was started (Month 19). A combination of moderate-fat dry and wet hypoallergenic, gluten-free diets with low RSS (diet 4a; 3.3 g fat/kg^0.75^) was introduced. There was no prior test for anti-gliadin and anti-transglutaminase A2 antibodies and to evaluate the effect of the diet on the neurologic symptoms, the ASD therapy was not altered at first, and the owner was advised to keep a diary of the neurologic episodes (Supplementary Table S1).

Three and a half months later (Month 23), the dog came for a follow-up consultation in which the owner reported four PD episodes after the onset of the diet trial. Two of those episodes were observed when a commercial dog treat (Dentastix^®^) was fed, and the other two were associated with accidental ice cream intake. Since there was an overall improvement in the PD episode frequency (Supplementary Table S1), a slow decrease in ASD was started. To avoid withdrawal epilepsy by the quick reduction of the ASD, the drugs were decreased by 25% per month. At that moment, the dog's BW had increased by 10% and BCS was evaluated as 6/9, therefore the calorie intake was decreased by 4%. Additionally, the owner requested a treat option, and a hypoallergenic, low-RSS, moderate-low-fat (3.62 g fat/100 kcal) treat was combined with the diet as 4.4% of the dog's daily calorie intake (diet 4b; 3.1 g fat/kg^0.75^).

During the first 2 months of the reduction of the ASD, the dog presented two PD episodes; the first one, 3 days after the first reduction of the medical treatment (Month 23); and the second one, one day after tapering the medication (Month 24). In the last month (Month 27) of the reduction of the medication the dog again presented a PD episode, but this was short-lived and since then the dog had remained symptom-free.

Three months later (Month 30), due to the unavailability of the prescribed commercial wet food, the owner contacted the nutrition service requesting another commercial wet food option. After careful evaluation of the commercial diets available on the market, no option was found to be suitable for all the criteria (moderate-low fat, low RSS, hypoallergenic and gluten-free). To have the lowest impact on the diet, a hypoallergenic and gluten-free wet food from the same brand, but specifically made for cats, was advised for a short period and only until the initially prescribed canine wet food was available again. Both wet food products had the same ingredient list, but they differed in nutrient composition. Since the cat's wet food was higher in fat, the dry and wet food ratio was changed, aiming at 3.5 g fat/kg^0.75^ (diet 5). Moreover, the owner was informed that the prescribed diet could change the targeted urinary pH and therefore, should not be used in the long term. Immediately after starting the new diet, the owner reported that the dog had two PD episodes followed by vomiting. The cat's wet food was then removed from the feeding plan and the previously advised diet (diet 4b) was resumed. Since then, no other neurological, gastrointestinal, or urinary clinical signs have been observed (Month 35). After that, the follow-up contact was reduced, but the owner was contacted again in Month 37 and small neurologic episodes were reported, those were short-lived and the owner did not record the dates. The timeline with all the events can be seen in Figure 1.

## 3. Discussion

Obesity is considered a predisposing factor for uroliths (12). The current obese patient developed CaOx stones, which were surgically removed and prevented by nutritional management as stated in literature (13–15). However, after the CaOx treatment, the patient presented a neurologic episode after remaining PD episode-free for 2 years and was hospitalized with presumed pancreatitis. Although the advised diet (diet 2) had low RSS, aiming for the decrease of the CaOx supersaturation, it also had higher fat content (+245%) than the dog's previous diet (diet 1). Due to the patient's history of use of ASD and its possible associated risk of pancreatitis (7–9), the higher fat intake was likely the cause of the gastrointestinal symptoms. Nonetheless, although serum amylase and serum lipase activity concentrations have limited value in the diagnosis of pancreatitis, due to their low sensitivity and specificity (16–18), based on the dog's medical history, symptoms, and physical examination, pancreatitis was strongly suspected. Additionally, CaOx preventive diets should be cautiously used whenever it is combined with medical treatment since the urinary pH can influence the rate of excretion of several drugs, including phenobarbital. The pH alkalinization increases urinary excretion of this drug and shortens its elimination half-life (19), decreasing its efficacy. Moreover, higher sodium intake increases potassium bromide excretion, decreasing its effectiveness (20). Therefore, the occurred neurological episode was potentially related to the lower efficiency of ASD after the dietary change.

Due to all the nutritional concerns and the risk factors from the different diseases (obesity, suspected pancreatitis, and CaOx), a weight loss program was firstly aimed at and successfully achieved without overlooking pancreatitis and CaOx stones. Changes in diet and body composition (obesity or weight loss) alter the pharmacokinetics of phenobarbital and adjustments in dose may be needed (21). At that moment the dog was well controlled on its PD episodes, therefore, no changes in the medical and nutritional therapy were advised. One year later, a neurological evaluation was performed after the increase in the PD episodes' frequency, followed by an unsuccessful attempt to increase the ASD dosage, and PD was diagnosed.

Canine PD is a movement disorder characterized by recurrent episodes of abnormal and involuntary self-limiting movements (1, 22). Although reports on dogs have increased in the past years, it remains important to differentiate it from epileptic seizures (11). In this case, the dog was first assumed to have epilepsy, and this led to long-term treatment with ASD. Most PD cases do not respond to ASD (5, 23–25), and only a few cases have been described to reduce the episodes' frequency upon medical treatment (26, 27). However, the presented patient remained episode-free for 2 years during the ASD therapy and this was potentially the reason for its misdiagnosis. The diagnosis of PD is based on the owner's description and video evaluation of the events. The most important differences between epileptic seizures and PD episodes are the preservation of consciousness, the longer duration of episodes (< 1 min vs. >1–5 min), and the lack of a post-ictal phase in PD cases (1). All of these features were seen in the reported case and confirmed the diagnosis of PD. In veterinary medicine, the most recognized form of PD episodes is paroxysmal non-kinesigenic dyskinesia, where the episodes are occurring spontaneously at rest (1). This type was suspected in the presented case (1, 28–31). Videos of the neurologic episodes showed episodes of dystonic movements. Dystonia-like movements are often seen in dogs with PD and are defined as sustained or intermittent involuntary contractions of a group of muscles producing abnormal movements, postures, or both (1, 20, 22).

PD has been described in different breeds (1), including the paroxysmal gluten-sensitive dyskinesia (PGSD) in Border Terriers, which often display concurrent dermatological and/or gastrointestinal signs (3–6, 11). Evidence shows partial or complete resolution of episodes after feeding the dogs with a gluten-free diet (2–6), however, scientific evidence is low. Although a positive response to gluten-free diets was seen in those case reports (2–6), a complete diagnosis of adverse reaction to food following the gold standard was not performed. For its diagnosis, serological tests (immunologic response against transglutaminase A2 and gliadin) were previously described as diagnostic markers for PGSD in Border Terriers (6), however, those tests were not formally validated for other dog breeds and are still limited in veterinary medicine. Additionally, although those markers may be an interesting tool, their results should be cautiously interpreted as they indicate if the animal was or was not previously in contact with gluten and it should not be assumed that high levels of those markers are diagnostic of PGSD. Nonetheless, it can be a useful marker to monitor compliance with a gluten-free diet therapy by testing it before and after diet change (6). For that reason, up to now, a diet trial is considered the gold standard to diagnose adverse reactions to food (32), including an adverse reaction to gluten. This trial involves the resolution of the symptoms after an elimination diet trial, followed by the reoccurrence of the symptoms after a challenge trial with the prior diet (32). For the elimination trial, a commercial or home-prepared novel protein diet or a commercial hydrolyzed protein diet should be consistently fed to the dog for 12 weeks or as soon as the symptoms ameliorate (33). However, the evaluation of neurological improvement can be difficult in a short period since animals with PD can stay several weeks/months without presenting another episode (34). Therefore, a long-term follow-up and a consistent diary can help interpret the results. Despite the lack of scientific evidence on the role of gluten on PD, in the current case, it was suggested to the owner to follow a gluten-free elimination diet trial as a first treatment approach and to re-evaluate the dog's symptoms after its completion. Due to the history of CaOx and the difficulty in controlling urinary pH with home-cooked diets, a commercial gluten-free diet with low RSS was preferred and recommended. In addition, to limit the risks of cross-contamination that over-the-counter gluten-free products may impose (35), a gluten-free hypoallergenic commercial diet with hydrolyzed protein was advised.

Although the standardized diagnostic trial, including the challenge phase, was not formally completed, in the current case, improvement of the symptoms was seen and the onset of the episodes after food indiscretion with a treat containing gluten was used as an accidental challenge. This was in agreement with previously PGSD documented cases (3–6, 11), as an improvement in the dogs' neurologic symptoms was observed after putting them on a gluten-free diet. Nonetheless, after those dogs (2–4, 6) clinically improved, there was no challenge with a gluten-containing diet, consequently, no confirmation of the suspected diagnosis.

In this case, although a causal relationship between a gluten-free diet and the management of its neurological symptoms is not 100% confirmed, the patient's clinical improvement and deterioration after an unintended challenge are supportive of a diet association. The re-challenge of the dog with gluten, documenting the recurrence of symptoms, would be the standard way to prove it. Nonetheless, it might not be strong enough to support such association, since the dog also presented symptoms after changing to another gluten-free diet (diet 5). Although the owner was convinced that the dog did not eat any other food besides the advised diet, food indiscretion cannot be entirely excluded, as it was observed at the beginning of the dog's diet trial (treat feeding with commercial pet food treats and human food). As stated before, the use of the serological tests (immunologic response against transglutaminase and gliadin) would have been a good option to ensure that the dog was being exclusively fed a gluten-free diet (6), however, since the dog was not tested before the start of the diet, testing the dog at this moment would be unreliable. Nonetheless, the increase in the fat intake from 3.1 to 3.5 g/kg^0.75^ could have been the cause of the vomiting and subsequently, the associated stress (5, 28, 29, 36) could have triggered the neurologic signs. The same symptoms were also noticed when the dog was hospitalized with suspected pancreatitis, presenting a PD episode after remaining episode-free for 2 years. Although we have previously mentioned that this PD episode could have been due to a decrease in ASD efficiency, it is also possible that this episode was triggered by the stress from the onset of gastrointestinal symptoms. This could be explained by the link between the nervous system and gut (gut brain-axis) (37, 38), and the possible interaction between those and the exocrine pancreas (39, 40). Finally, after a few months of neurological episodes remission, the owner was contacted again and reported the occurrence of short-lived episodes, but since the owner's commitment to keeping a diary was low, no associations could have been evaluated at this point.

Some important factors should be emphasized in the presented case report. Firstly, this patient was misdiagnosed and was long-term treated for epilepsy, increasing the risk of chronic pancreatitis (7–9, 20). Secondly, for patients with several medical conditions, nutrition management should be aimed at an individual basis, taking into consideration medical history, treatment, dietary history, and risk factors of each disease. In this case, after the removal of the CaOx stones, a general diet for uroliths prevention (diet 2) was advised by the primary veterinarian, which was not meeting all the criteria (key nutrients) for the prevention of pancreatitis and the treatment of obesity. Due to the patient's increased risk of pancreatitis, the use of an inadequate diet with a high-fat content (diet 2) was the possible cause of the gastrointestinal symptoms. Finally, although it has been hypothesized that gluten intolerance/allergy is associated with the onset of neurologic symptoms, this has not been proven yet. Other dietary proteins could be possible culprits as advised diets for PD patients are generally not only gluten-free but also hypoallergenic. In future investigations, it might be interesting to combine the use of serological tests (immunologic response against transglutaminase A2 and gliadin) with an elimination diet trial. The use of both diagnostic tools can lead to a better understanding of the etiology of the disease.

To the authors' knowledge, this is the first case report of PD in a German Spitz as well as the first PD case with a complete nutritional history and a long follow-up. Despite several limitations that a case report imposes, as well as the lack of information regarding the etiology of the disease, it was interesting to see the clinical improvement of this patient after nutritional management. This showed the importance of a precise nutrition anamnesis, a detailed nutritional evaluation of the previous diets, the continuous follow-up of the patient, the owner's compliance, and the adaptation of diet and/or nutrient intake for the prevention of clinical symptoms, especially for patients suffering from several medical conditions.

## Data availability statement

The original contributions presented in the study are included in the article/supplementary material, further inquiries can be directed to the corresponding author.

## Ethics statement

Ethical review and approval were not required for the animal study because this case report describes the clinical treatment of a privately owned animal. Written informed consent was obtained from the owners for sharing the videos and the description of this case.

## Author contributions

CB was the veterinarian directly managing the nutrition issues of this case with NR-S's support. MHer was the veterinarian directly managing the neurologic symptoms of this case with FV's support. MHes and SB supervised and mentored CB. CB wrote the report with assistance and feedback from MHer, NR-S, FV, SB, and MHes. All authors contributed to the article and approved the submitted version.
